# Sport and Eating Disorders - Understanding and Managing the Risks

**DOI:** 10.5812/asjsm.34864

**Published:** 2010-06

**Authors:** Alan Currie

**Affiliations:** Newcastle General Hospital, United Kingdom

**Keywords:** Eating disorders, Sport, Athletes, Prevention, Screening

## Abstract

There is strong and consistent evidence that eating disorders are prevalent in sport and especially in weight sensitive sports such as endurance, weight category and aesthetic sports as well as jumping events. These illnesses are not only common but lead to significant physical and psychological morbidity and impaired performances.

Sports organizations, and by extension the professionals whose job it is to help and support athletes, have important roles in dealing with these conditions. Preventative practices can be adopted if there is an understanding of how the sports environment contributes to the development of eating disorders. Some disorders can be difficult to detect especially in a sports environment and simple screening instruments are available. Athletes may also need help to access appropriate treatment whilst they are recovering.

In many sports prevention, screening and support programs have been developed for a variety of medical conditions or sportsrelated injuries. Similar programs should be developed for eating disorders.

## INTRODUCTION

Eating disorders are serious and common psychiatric conditions. Anorexia Nervosa is characterised by an intense fear of becoming overweight (despite being underweight), body image distortion and denial of low weight, refusal or inability (via disordered eating behaviours) to maintain normal body weight and amenorrhoea. Bulimia Nervosa is more common and the central features of this condition are regular and frequent food binges (often very large quantities and consumed in a rapid, out of control manner), typically followed by a compensatory purge (often self-induced vomiting) and there is a morbid dread of fatness. Sub-clinical forms of either disorder or a mixture of the two conditions are especially common and usually called Eating Disorder not Otherwise Specified (or EDNOS)^[1]^.

Although traditionally viewed as a ‘western’ disease driven by social and cultural pressures to conform to an ideal and unrealistic body-type^[2]^, these conditions are in fact complex, multi-factorial and found across all societies and cultures. Their incidence in ‘non-western’ cultures is not trivial^[3, 4]^ and includes significant prevalence rates and morbidity in sporting populations^[5]^.

It is not surprising to find that these conditions are found in sportsmen and women. The disorders are especially common in sports where weight has a significant effect on performance. There are three principal reasons for this. Firstly, in endurance sports such as long-distance running, leanness is related to performance for obvious physiological reasons. Runners who are several kilogrammes over their optimum performance weight will perform less well. Secondly, in weight category sports such as judo, boxing and wrestling, athletes will not be allowed to compete if their weight is above the upper limit for that category. Athletes have had to return from the Olympic Games without competing for this reason. This can create considerable pressure to achieve the necessary weight loss and often in a very short period of time. Thirdly, in sports such as gymnastics and high board diving, an aesthetic evaluation is attached to a particular body composition which is then promoted and encouraged in competitors. Major studies have consistently reported higher prevalence rates in these groups of sports^[6–9]^.

Prevalence studies in sports populations are often difficult to interpret, especially if they are surveys of heterogeneous groups of athletes of mixed standard, if no control group is included and if self-ratings or screening questionnaires are used in preference to clinical interviews and more detailed evaluations^[10]^. The largest study to address all three of these areas^[9]^ found a significantly increased prevalence of all eating disorders in all three groups of sports (endurance, weight category and aesthetic sports) in female competitors (Table 1). Male competitors also had a significantly increased prevalence in so-called ‘antigravity’ sports such as ski-jumping and other jumping events where excess body weight has a disadvantage (Table 2).

**Table 1:** The prevalence of eating disorders in elite sportswomen

	Anorexia No (%)	Bulimia No (%)	EDNOS No (%)	Total No (%)
**All sportswomen (n=572)**	11 (2)	36 (6)	68 (12)	115 (20)
**Control subjects (n=574)**	1 (0)	17 (3)	34 (6)	52 (9)
**Aesthetic (n=52)**	6 (12)	8 (15)	8 (15)	22 (42)
**Weight category (n=53)**	0	6 (11)	10 (19)	16 (30)
**Endurance (n=102)**	4 (4)	10 (10)	10 (10)	24 (24)

Adapted from:- Sundgot-Borgen & Torstveit, 2004

Anorexia = Anorexia Nervosa (DSM-IV classification)

Bulimia = Bulimia Nervosa (DSM-IV classification)

EDNOS = Eating Disorder Not Otherwise Specified (DSM-IV classification)

Percentages are to the nearest whole number

**Table 2:** The prevalence of eating disorders in elite sportsmen

	Anorexia No (%)	Bulimia No (%)	EDNOS No (%)	Total No (%)
**All sportsmen (n=687)**	0	17 (2)	38 (6)	55 (8)
**Control subjects (n=629)**	1(0)	1 (0)	1 (0)	3 (0)
**Antigravity (n=37)**	0	1 (3)	5 (14)	6 (16)
**Weight category (n=79)**	0	7 (9)	7 (9)	14 (18)
**Endurance (n=149)**	0	5 (3)	9 (6)	14 (9)

Adapted from:- Sundgot-Borgen & Torstveit, 2004

Anorexia = Anorexia Nervosa (DSM-IV classification)

Bulimia = Bulimia Nervosa (DSM-IV classification)

EDNOS = Eating Disorder Not Otherwise Specified (DSM-IV classification)

Percentages are to the nearest whole number

In some respects, the prevalence of eating disorders is not the central issue. The high prevalence in sports is certainly a cause for concern but these conditions are potentially serious for any sufferer. Mortality rates of 5.9% have been reported^[11]^ with standardised mortality rates of between 3.6 and 9.9^[12]^ and medical complications are frequent and extensive^[13]^. Those who work in the sports field have an additional concern, for even if the more serious morbidity can be avoided, an athlete with an eating disorder can expect to be more prone to injury, and to have a shorter sports career that is troubled by inconsistent performances^[14]^.

## PREVENTION

If the problem is to be addressed then there needs to be an understanding of how the sports environment might contribute to an increased risk. It is knowledge of additional risk factors that leads to the development of preventative strategies in the world of sport. Sports organisations, sports governing bodies and those professionals who work with athletes have a responsibility to develop and implement good preventative practices. Fortunately (and self evidently) there is considerable overlap between those practices that keep athletes healthy and those practices which improve and sustain their performances.

Sportsmen and women develop eating disorders for the same reasons that others do. There may be individual genetic and/or psychological vulnerability, socio-cultural pressures relating to diet, food and body image and non-specific psychological stressors which can act as trigger events. The sports environment can add to these risks in several ways especially in endurance, weight category and aesthetic sports. Other factors have also been described, for example the revealing nature of much sports clothing, the intense competitiveness of sports participants which can extend to ‘competitive thinness’^[15]^, specialising in one sport at an early age and sudden increases in training volume^[16]^.

A broad ranging (and successful) preventative strategy would be one that addressed many or all of these sports specific factors whilst still allowing athletes and their coaches to strive for excellence and explore the margins of human performance.

To achieve these aims it is necessary to consider the nutritional support required by athletes to maintain health and support performance. There needs to be an agreed understanding of what constitutes the athlete's optimum performance weight. For weight category sports this is externally defined. Athletes who consistently struggle to make weight in these sports may need additional support with their nutritional strategies or to consider competing in a different weight category.

Some athletes will have an optimum performance weight that is different for training and competition. This is common in elite endurance cycling. Athletes who need to manipulate their weight should be encouraged and supported to use low risk strategies. Weight loss is least risky if it is pursued under supervision, gradually and with an agreed target in mind. Athletes have special needs regarding their dietary requirements and making appropriate quantities of the right foodstuffs available to them can be a challenge both at training camps and during competitions. Weighing athletes in public can reinforce ‘competitive thinness’ and lead athletes to make unfavourable comparisons with their team mates. This in turn can promote disordered eating behaviours. It is best avoided.

Sports coaches can also contribute to reducing the eating disorder risk. A supportive environment which encourages athletes to make the best of themselves is to be preferred over one where criticism and bullying (especially in relation to weight and body composition) are the norm.

UK Sport has produced a comprehensive guideline detailing the nutritional and coaching practices to be adapted when managing the sports-specific eating disorder risks^[17]^. Other large sports organisations have issued guidelines and position statements including the American College of Sports Medicine^[18]^.

## SCREENING, IDENTIFICATION AND ACTION

Eating disorders are not easy to identify. Whilst the physical manifestations of anorexia nervosa may be clearly evident to the observer, the related disordered eating behaviours are likely to be kept secret by the sufferer and denial is a common feature even in advanced cases. Many sufferers of bulimia nervosa are of entirely normal physical appearance and as with anorexia nervosa the associated eating behaviours (in this case bingeing and purging) will be kept hidden.

In the world of sport there are additional identification difficulties. In many sports the sufferer's fellow athletes will be lean compared to population norms. An athlete whose body composition results from anorexia rather than athleticism will be harder to spot ^[15]^. Sportsmen and women will also be expected and encouraged to eat in unusual ways. For most this will simply be another aspect of the pursuit of sporting excellence. For some it will herald a progression from unusual eating patterns (which are normal in a sporting population) to more clearly disordered and pathological dietary measures. It can be difficult to distinguish ‘athletic’ from ‘disordered’ eating and there is no clear cut-off point. Some distinguishing characteristics have been described^[14]^. ‘Athletic’ eating is more likely to be directed towards improved performance rather than weight loss or altered body shape. There is more emphasis on what needs to be eaten than what is forbidden and eating habits ‘normalise’ in the close season and on retirement. In some sports the measurement of body mass index or body weight can be an unreliable indicator of potential eating disorders. A highly muscled individual will be heavy (with a correspondingly elevated body mass index) although lean. If he/she begins to lose weight he/she may not meet all the diagnostic criteria for anorexia nervosa until the condition is advanced and detection of the condition may be delayed unless a watchful eye is kept on absolute weight, progressive changes in weight and dietary intake.

In sporting populations it is important to look hard for the presence of disordered eating, not simply because eating disorders are potentially serious conditions but also because they can be difficult to detect. Many sports organisations promote regular screening for eating disorders and simple, reliable and validated screening instruments are available, for example the 5-item ‘SCOFF’ questionnaire^[19]^.

Screening programmes are only helpful if there is a clear understanding of what follows. An athlete with a possible eating disorder should be approached early, directly, supportively and confidentially^[20]^. In the first instance a more detailed medical and if necessary psychiatric evaluation is required. This should clarify if a problem is present, its severity and whether any immediate action is required. Some more minor problems can be dealt with by providing basic nutritional advice and support^[9]^ but all problems will benefit from prompt action.

Accessing treatment, even in highly resourced health economies is often difficult. Specialist treatment services are often some distance away and/or have long waiting lists. As with all other medical conditions it is better to prevent than to cure. This requires a sports environment where the risks are acknowledged and managed and where good preventative practice is the norm.

## RECOVERY

Whilst some eating disorders can become chronic debilitating conditions^[21]^ many sufferers can expect to make a good functional recovery^[22, 23]^ especially if problems are detected early and acted upon promptly.

An athlete who is making good progress in therapy may be in a position to resume sports participation. There is a clear parallel here with the athlete returning to sport after a serious injury. Returning to a light training load is the first step and as recovery progresses the training load increases until eventually the athlete is ready to return to competition. A graded return like this can serve as a powerful reward for making progress in therapy^[15]^. As with injury rehabilitation, close cooperation between the therapist/physician and coach/athlete is essential. There are four factors to be considered when planning a rehabilitation program^[24]^. In order of priority, they are medical stability (including any electrolyte abnormalities, anaemia, low bone density etc), nutritional stability (a minimum requirement is that nutrition is sufficient to maintain weight despite the increased energy expenditure), abstinence from disordered eating behaviors and consideration of the psychological stresses that may be present in the sports environment and which may contribute to a relapse of the condition. It is occasionally necessary to consider offering athletes support to leave the sports environment. It may simply be too toxic and too likely to promote relapse or the disorder itself so severe that return is inadvisable.

Difficult and painful decisions such as this can be made easier when there is an open and trusting working relationship between athletes, coaches and clinicians and where processes are explicit from the outset. A coach and athlete should be in agreement about what each expects from the other. A professional athlete under a contract should be clear about what medical support will be provided, in what circumstances and what is expected in return. This might include subjecting him or herself to health screening and being expected to take advantage of health care when it is offered. The criteria to be used if an athlete is not allowed to train or compete on health grounds may need to be stated. Applicable criteria include acute medical conditions that make it unsafe to compete or more chronic conditions that are incompatible with sustained training or competition. Many contracts are also explicit about the circumstances under which the contract will end. This is usually a consequence of deteriorating performances, ill health or injury (or a combination of the three). An athlete should expect that all health problems (whether injury, eating disorders or any other illness) are treated equally. It would be unhelpful and unjust to apply different criteria to exclude an athlete with an eating disorder.

## CONCLUSION

Eating disorders are common and potentially serious conditions which affect both health and sporting performance. The sports world is familiar with how to deal with the risks of sports injury. This includes developing good practice in prevention, screening programs and helping athletes to get the right treatment and support when they need it. A similar framework should be adopted for eating disorders.
